# Cardioprotection and Thyroid Hormones in the Clinical Setting of Heart Failure

**DOI:** 10.3389/fendo.2019.00927

**Published:** 2020-01-28

**Authors:** Francesca Mastorci, Laura Sabatino, Cristina Vassalle, Alessandro Pingitore

**Affiliations:** ^1^Clinical Physiology Institute, CNR, Pisa, Italy; ^2^Fondazione G. Monasterio, CNR-Regione Toscana, Pisa, Italy

**Keywords:** cardioprotection, heart failure, epigenetic, thyroid hormones, subclinical thyroid disorders

## Abstract

Ischemic heart disease is the main cause of morbidity and mortality worldwide and is becoming more widespread with population aging. Cardioprotection is a dynamic process characterized by mechanisms related to myocardial damage and activation of protective factors. Targeting these processes could be attractive as a new therapeutic strategy in the evolution of post-ischemic heart failure (HF). In this context, the role of thyroid hormone (TH)-mediated cardioprotection is supported by a number of findings regarding the modulation of neuroendocrine systems, inflammatory and oxidative stress status, pro-survival intracellular pathways, and epigenetic factors, its effects on cardiac angiogenesis, structure, and function and on the preservation of mitochondrial function and morphology, and its beneficial effects on cell growth and redifferentiation. Moreover, the numerous effects of TH on the heart involve genomic mechanisms, which include cardiac differentiation during the perinatal period and non-genomic action, directed toward the maintenance of cardiovascular homeostasis. This evidence suggests that there is an opportunity to treat HF patients with TH. This review is mainly focused on the clinical evidence of the role of the thyroid system in the complex setting of HF.

## Thyroid Hormones in the Setting of Heart Failure

Heart failure (HF) represents a progressive chronic degenerative disease, starting with systolic or diastolic dysfunction of the left ventricle and evolving toward systemic disease, in which other organ dysfunctions and systemic responses are involved. The main cause of HF is ischemic coronary artery disease, with or without myocardial infarction, as a consequence of coronary blood flow reduction, myocardial necrosis, and the ischemic remodeling process. The clinical and social impact of HF is relevant considering its incidence, severity, and social costs (1–3) and also in association with progressive aging of the worldwide population (4–6).

**Figure 1:**
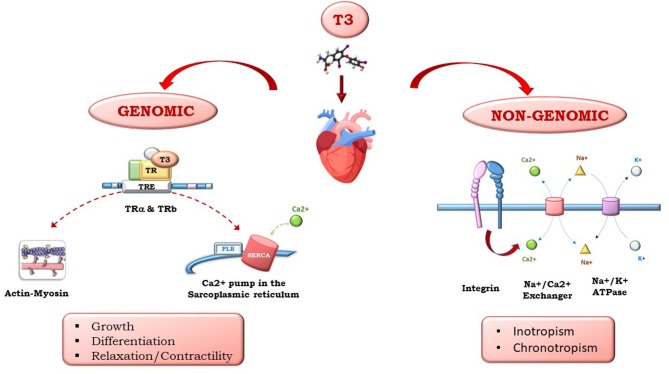
Examples of genomic and non-genomic TH effects.

Triiodothyronine (T3) and thyroxine (T4), produced by the thyroid gland, have multiple effects on the heart. Whether circulating T4 originates from the thyroid, T3 is produced peripherically by T4 5′-monodeiodination. Thyroid hormones (THs) influence both diastolic and systolic functions and have relevant effects on cardiac morphology and structure, coronary vasculature, cell metabolism, and cell protection, growth, and differentiation. As shown in Figure 1, the multiple effects of TH on the heart are mediated by different signaling pathways that have been clustered into genomic and non-genomic actions: THs regulate gene expression through specific nuclear α and βTH receptors (TRs) and regulate genes involved in metabolism, cell growth and differentiation. THs also exert non-genomic actions via interactions with cytoplasmic and membrane-associated TRs, such as integrin αvβ3, that mediate TH action on the transport of ions across the plasma membrane and glucose and amino acid transport. THs act directly on the myocardial structure, regulating the interstitial collagen content within the myocardium, favor the development of coronary angiogenesis, thus increasing coronary flow reserve, and regulate cardiac function through chronotropic, inotropic, and dromotropic effects. Furthermore, THs also act through synergistic actions with inflammatory and neuroendocrine systems, as well as oxidative stress machinery, through direct action on the mitochondria. The high level of TH signaling integration with other systems is particularly relevant in HF. In fact, TH has an initial protective role, and its continuous activation results in toxic effects on all the systems implicated in HF pathophysiology.

## Thyroid and Heart Failure in the Clinical Setting: the Prognostic Impact of TH Abnormalities

Hypothyroidism is the TH disorder that is most studied in experimental and clinical settings of HF. The studies are mainly focused on the prognostic role of hypothyroidism and on the effects of TH replacement therapy on cardiac function and clinics. The mild hypothyroid alterations investigated in HF clinical settings are subclinical hypothyroidism (SCH) and low triiodothyronine syndrome (LT3S). SCH is defined by 4.5 mIU/L ≤ TSH ≤ 20 mIU/L, with a normal T4 concentration. Actually, a TSH value of 10 mIU/L is the suggested cut-off for substitutive thyroid therapy. LT3S is defined as low T3 circulating plasma levels in the presence of normal thyroxine and TSH levels.

In the meta-analysis of Wang et al., LT3S incidence was higher in HF patients than in those with acute coronary syndrome, suggesting LT3S as a marker of chronic disease (7). In the context of acute decompensated HF, LT3S, and SCH are also considered markers of clinical severity, left ventricular dysfunction, and hemodynamic impairment (8–11). Accordingly, in the study of Rothberger et al., in which 137 patients with acute decompensated HF were enrolled, those with LT3S (48%) had a greater need for mechanical ventilation (12). Similarly, patients with SCH showed a more impaired clinical status. In the study of Sato et al., SCH was associated with lower oxygen consumption and higher mean pulmonary arterial pressure in comparison to euthyroidism (13).

In the clinical studies, there are conflicting results regarding the prognostic relevance of TH abnormalities, reflecting the heterogeneity of the population enrolled in terms of HF cause, sex, age, race, degree of TSH suppression, and duration of follow-up, modality of acquisition of the data and their fullness, time of TH sampling, and time of patient enrolment. Indeed, available studies include patients with both ischemic and non-ischemic HF and patients with stable and acutely decompensated HF. Furthermore, the events considered included cardiac and overall death, as well as hospitalization and arrhythmias. Finally, the chosen age range was variable (37–75 years). Moreover, in the study of Okayama and Sato, patients with normal left ventricular function were also enrolled (14, 15). The study of Mahal et al., showing that in hospitalized HF patients with SCH there was no increase in mortality and major morbidity, was a retrospective population study in which the data were obtained from the Nationwide Inpatient Sample (NIS), using validated ICD-9CM (classification of diseases, 9th edition, clinical modification) codes for the identification of patients with HF and other comorbidities (16). Among 143.735 patients, half of them were above 80 years whereas a low proportion of patients were below 60 years of age (11%). This is a relevant factor if we consider that TSH values increase with aging, and this justifies the use of different reference intervals in old subjects, in particular in subjects 60 years old and older (17). Therefore, the high limit of TSH >4.0 mIU/l to define SCH can be restrictive in old people, and thus a larger TSH normal reference range should be used to avoid misdiagnosed SCH. This evidence should also partially explain the absence of clinical benefit from levothyroxine in older patients in terms of reducing cardiovascular events and mortality (18).

In the study of Frey et al., the absence of prognostic weight of SCH might be a consequence of the fact that TH replacement therapy was not an exclusion criterion of the study (8). Indeed, 27% of SCH patients were treated with levothyroxine. Usually, information on the persistence of the altered thyroid status, in the follow-up, may further increase prognostic stratification. In fact, when evaluated in the follow-up, the development of TH disorders was observed in patients with chronic stable HF without evidence of previous TH abnormalities (LT3S 12.5%, SCH 10.4%, overt hypothyroidism 6.2%), suggesting that it may be an important factor for HF progression (19).

It may be important to consider TH abnormalities in the context of a multiparametric approach in the prognostic stratification of HF patients, in which different clinical, biohumoral, and functional data are included. In this perspective, the study of Li et al. (20) showed that SCH was not an independent predictor of all-cause mortality after adjusting for other confounding factors, although patients with SCH had a higher incidence of overall mortality. On the contrary, the study of Wang et al. showed that FT3 was the stronger predictor of cardiac events, together with the extent of necrosis (21). In addition, cardiac death was significantly higher in patients with HF and LT3S in comparison with patients with a similar left ventricular ejection fraction but normal total T3. This finding indicates that LT3S is an independent predictor of mortality, adding prognostic information to conventional clinical and functional cardiac parameters, such as left ventricular ejection fraction (22). Moreover, the negative prognostic stratification is improved by combining measurement of brain natriuretic peptide (BNP; a hormone secreted by cardiomyocytes in response to stretching derived by increased ventricular blood volume, and a validated HF biomarker) with the presence of LT3S both in acute decompensated and in chronic compensated HF (23, 24). In another study by Sato, LT3S was associated with high cardiac and overall mortality, accompanied by high central venous pressure, lower nutritional status, and impaired exercise capacity (15). Moreover, the prognostic relevance of TH abnormalities may also be influenced by the fact that TH abnormalities occur in co-morbidities frequently observed in HF. In particular, in HF patients with hypothyroidism, renal insufficiency was significantly worse than in patients with normal thyroid function (25, 26). Also, in hemodialyzed patients, LT3S was strongly associated with cardiac death, and TH replacement therapy attenuated the rate of decline in renal function in chronic renal failure patients with SCH (27, 28). Therefore, although there are no relevant data on the potential association among TH abnormalities, HF co-morbidities, and HF, it is conceivable that these associations could result in a vicious cycle, leading to additive and detrimental effect on the clinical conditions and prognosis of HF patients.

## TH Treatment in HF Patients

Several clinical studies report different data on TH replacement therapy regarding methodologies using short-term and long-term TH replacement therapy, T3 or T4 at different doses, and modality of administration. In addition, there are differences in the population cohorts in which these treatments are adopted, including HF patients with a normal or abnormal TH profile or with stable or unstable HF (29–37). Thus, conclusions are far from being definitive and widely shared by the scientific community.

### Thyroxine Treatment

In stable chronic HF patients (*n* = 10) with idiopathic dilated cardiomyopathy, oral administration of T4 at a dosage of 100 μg/day for 1 week and 3 months (30, 31) was well-tolerated, inducing increased cardiac function and reducing systemic vascular resistance. Furthermore, the administration of dobutamine (10 μg/Kg/min) (a direct-acting inotropic agent and an adrenergic agonist that primarily stimulates the β1-adrenoceptors, increases myocardial contractility and stroke volume, also reducing peripheral vascular resistance and ventricular filling pressure) improved cardiac output and heart rate in T4-treated patients (*n* = 10), suggesting enhanced cardiac adrenergic sensitivity, in line with experimental data showing β1-adrenergic up-regulation via TH (38). Moreover, in the study of Curotto Grasiosi, T4 administration in HF-SCH patients (*n* = 163) improved functional capacity and physical performance (32). In acute decompensated HF patients (*n* = 20), unresponsive to conventional inotropic therapy and intra-aortic balloon counterpulsation, the positive hemodynamic effect of intravenous T4 administration (20 μg/h) was documented and maintained for enough time to complete surgical treatment consisting of heart transplantation or left ventricular device (33).

### Triiodothyronine Treatment

In patients with HF, T3 administration has been applied both orally and intravenously (29, 34–37). In the study by Hamilton et al., patients (*n* = 23) with advanced HF (NYHA functional class III-IV) and low T3 levels and/or elevated plasma reverse T3 (rT3) values who received a high dose of T3 (T3 bolus of 0.7 μg/kg followed by 6-12 h T3 infusion to a total dose of 1 or 2 μg/kg) had a significant increase in cardiac output and a reduction in systemic vascular resistance (34). In another two studies, constant infusion of L-T3 induced a progressive reduction in systemic vascular resistance and an increase in ejection fraction and cardiac output (29, 35). Moreover, in the same type of HF population (*n* = 10), a 3-day continuous T3 infusion induced an increased stroke volume and end-diastolic volume (29) without an increase in myocardial oxygen consumption. The increased end-diastolic volume can be considered to reflect the recruitment of residual ventricular filling reserves due to the effects of T3 on diastolic relaxation (39, 40). These results fit well with those observed after normalization of the thyroid state in patients with mild primitive hypothyroidism and without cardiac diseases. In these patients, left ventricular stroke volume, ejection fraction, and cardiac index significantly increased after synthetic TH replacement therapy, while blood pressure and heart rate did not change (41). Moreover, T3 infusion induced a reduction in the vasoconstrictor/sodium-retaining norepinephrine and aldosterone concentration, and in NT-proBNP (amino terminal fragment of brain natriuretic peptide precursor) levels, resulting in a circulating neuroendocrine biomarker profile improvement (29). By contrast, in 3 months of oral T3 therapy in HF patients (*n* = 13) with stable chronic HF and low T3 levels, a lack of benefits in cardiac function and in neurohormonal changes was observed (36).

The contrasting results observed can be attributed to the fact that, in the first study, patients had a lower ejection fraction and higher NT-proBNP, indicating a more severe clinical status (29, 36); furthermore, the type of administration and T3 dosage (intravenous vs. oral) were completely different, determining the different changes in TH levels (29, 36). In addition, the stability of circulating T3 levels was guaranteed with continuous intravenous infusion, whereas it is improbable that the same result would be gained with only two daily T3 doses, administered orally (29, 36). In a more recent study (37), in which T3 was administered long-term (6 weeks) at a dose of 0.025 mg/day per os in 39 patients, the left ventricular ejection fraction was improved, whereas NT-proBNP and inflammation markers were reduced and, interestingly, exercise capacity, evaluated by 6-min walking distance, was increased. Importantly, these contrasting results may suggest that the selection of HF patients and the modality of administration as well as the TH dose are determinants for a more effective therapy in HF. Moreover, these results underscore an important endpoint of TH treatment, which is to restore and maintain levels of circulating TH and TSH within their respective reference ranges.

Currently, it is not established whether T3 or T4 can be used in HF patients, which of them is more efficacious, and at which doses. Although T4 administration may represent the more physiological treatment approach, due to the fact that T3 is derived mainly from the T4 to T3 peripheral conversion, an increasing body of data suggest that the use of T3 is more effective. In fact, peripheral T4 to T3 conversion is impaired in chronic diseases such as HF, and thus the administration of T3 may be overcome by this step. This is a central aspect, since the cardiovascular system responds mainly to T3. Nonetheless, experimental findings have shown that only combined T3 and T4 treatment guarantees euthyroidism in all tissues of thyroidectomized rats, including myocardium (42).

Since the goal of TH replacement therapy in HF patients is to restore and maintain euthyroidism, supraphysiological doses (“pharmacological” hyperthyroidism) should be avoided. Until results from large, randomized clinical trials are available to confirm long-term safety and efficacy, the suggested substitutive dose of T3 should not exceed 0.2–0.4 μg/kg per day (that is, 15–30 μg per day, divided into two or three administrations) and about 1 μg/kg per day for L-T4 (that is, 50–100 μg once daily).

## Thyroid and Cardioprotection

In general, the experimental studies showed the negative impact of altered thyroid metabolism on cardiac function, metabolism, cell protection, and surveillance, as well as on mitochondrial function and protection, and the reversibility of these alterations when restoring euthyroidism. This experimental evidence provides a strong indication of the potential role of thyroid hormones in cardioprotection.

The term cardioprotection includes all mechanisms contributing to heart protection by reducing or even preventing myocardial tissue injury and affecting multiple factors involved in cardioprotection (e.g., cytokines, cell growth, angiogenesis, and mitochondria). In this context, cardioprotection emerges as an attractive novel therapeutic strategy in the evolution of post-ischemic HF (43–45). There are several differences between acute and chronic HF myocardial damage. Acute ischemic HF myocardial damage is due to the abrupt occlusion of a coronary artery and reperfusion damage and ismore characterized by cellular death. In contrast, during chronic ischemic HF, a variety of remodeling processes and stimuli occur, which usually require up to several months (46), inducing changes in cardiomyocytes, extracellular matrix, and vasculature. The final result is the thinning of the infarct area and its expansion at the site of the necrotic border zone; hypertrophy and fibrosis of the remote zone likely occur as a direct response to increased wall stress (47).

**Figure 2:**
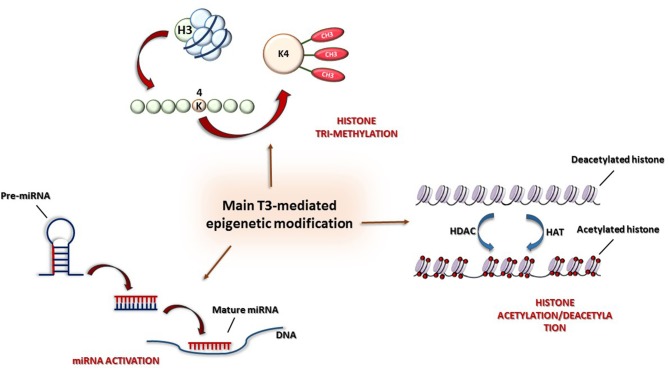
Main epigenetic mechanisms: (1) Histone modification by acetylation/deacetylation, (2) tri-methylation of lysine 4 in histone H3 (H3K4me3), and (3) post-transcriptional control by miRNAs.

Briefly, we report some experimental findings showing the multiple effects of TH on cardioprotection; these are illustrated in Figure 2.

### TH and Pro-survival Intracellular Pathways

The cardioprotective effect of TH depends on the regulation of prosurvival pathways, including the activation and function of the phosphatidylinositol 3-kinase (PI3K)/Akt and PKC signaling cascade, the expression, phosphorylation, and translocation of heat shock protein 70 (HSP70) and HSP27, and the suppression of p38MAPK signaling (48). In particular, 3-day T3 treatment after acute myocardial infarction (AMI) had an antiapoptotic effect, reducing myocyte apoptosis in the border area, via Akt signaling (39). Pantos et al. (49) also reported the same outcome, with decreased p38 MAPK activation. Recently, TH was found to have a dose-dependent effect on Akt phosphorylation (50). Mild activation of Akt caused by a replacement dose of TH resulted in positive effects, while further induction of Akt signaling by higher doses of TH was accompanied by increased mortality and activation of ERK, a kinase linked to pathological remodeling (51). This result suggests that the induction of pharmacological excess of thyroid function should be avoided, since it could have a negative impact on patients; therefore, the object of thyroid replacement therapy in HF patients should be the restoration and maintenance of euthyroidism.

### TH and Myocardial Interstitium

THs also regulate collagen interstitial matrix biosynthesis, and their alteration can induce remodeling of the interstitial collagen content (52). In thyroidectomized rats, pro α1 (I) collagen and pro α2 collagen mRNAs and relative proteins increased significantly in myocardium, as did transforming growth factor (TGF)-β1. The activation of this molecular signaling induces a pro-fibrotic process through miRNA signaling (53). The activation of TGF-β1 signaling is hampered by early and short-term T3 supplementation, as documented in a rat model of ischemia/reperfusion, and this was associated with a reduction in scar size and preservation of cardiac performance (54).

### TH and Coronary Vasculature

TH-induced cardioprotection is also exerted through the effect on coronary circulation. Hypothyroidism determines the reduction in arterial length and density, with the impairment of coronary vasodilation reserve, and this has been observed during hypothyroidism induced both by thyroidectomy and by propyl-thiouracil (55). The return to euthyroidism via T3 or T4 induced pro-angiogenic effects, with a restoration of arterial length and density (56). Similarly, in diabetic dysfunctioning heart, T3 treatment prevented the rarefaction of arteriolar resistance vessels with increased gene expression of TR-β, vascular growth factors (vascular endothelial growth factor A, VEGF-A), and endothelial nitric oxide synthase (eNOS) (57). TH-induced pro-angiogenesis is mediated by integrin αVβ3 that through the receptor site (S2) that binds both T3 and T4, activates extracellular regulated kinase (ERK) (58). Further, the expression of hypoxia-inducible factor 1 alpha (HIF-1a) through the interaction of TH with cytoplasmic TRβ and the activation of P13K signaling is another molecular circuit involved in T3 pro-angiogenesis (59).

### TH and Mitochondria

TH is associated with the upregulation of mitochondrial proteins with functional relevance. In particular, TH mitochondrial protection is mediated by the regulation of tumor suppressor p53, whose network is activated under stress conditions such as AMI and HF and enhances the mitochondrial pathway of cell death (60). More recently, correction of post-ischemic LT3S has been shown to downregulate the mitochondrial-targeted noxious effect of protein p53, possibly through the upregulation of miR-30a (61). T3 treatment counteracts the decrease in miR30a level, limiting the activation of p53 and the cascade leading to mitochondrial injury and cell death in the border zone of myocardial infarction (62). Interestingly, in the human setting of post-ischemic HF, the levels of p53-responsive miRNAs (miR-192, miR-194, and miR-34a) were associated with adverse post-ischemic remodeling, indicating that they have the potential to be predictive of future ischemic HF (63).

TH-induced cardioprotection can also be provided by interaction between the thyroid system and the inflammatory, neuroendocrine, and oxidative stress systems, which, in turn, have a key role in the pathophysiology and clinical progression of HF. Another new area for consideration is the epigenetic mechanisms of TH regulation, which could open up new therapeutic opportunities.

### TH and Inflammation

The so-called cytokine hypothesis highlights the importance of the dangerous effects of continuous activation of the inflammatory system, which favors the progression of heart damage and dysfunction, inducing apoptosis, mainly in myocytes and endothelial cells (64, 65). Experimental and human studies showed a cross-talk between TH and inflammation. In particular, Hajje et al. showed that propil-thiouracil-induced-hypothyroidism increased the plasma concentration of inflammatory markers C reactive protein (CRP) and cytokines (e.g., TNF-a and IL6) as well inflammatory gene markers (e.g., TGF-β1 and cTGF and Il1 and Mcp1). However, unexpectedly, the authors also observed a further increase in inflammatory markers despite improvement in cardiac function after the restoration of euthyroidism through T4 treatment, rendering these data difficult to interpret (66). Interestingly, in human cultured thyroid follicles, IL6 inhibits thyroid function, which might account for changes observed in LT3S (67, 68). Similarly, the administration of recombinant human IL-6 (rhIL-6) in animals resulted in an early decrease in serum T3 (69). The study of Lubrano et al. showed that, in patients with advanced HF, inflammatory markers TNFα, IL-6, and CRP correlated inversely with free T3, and this increase was significantly higher in patients with LT3S (free T3 < 2 pg/ml) (70).

### TH and Oxidative Stress

Elevation of oxidative stress (OxS; an imbalance between the generation of reactive radical species and antioxidant defense) has been demonstrated in every phase of HF development, and several biomarkers, such as those related to protein, lipid, or DNA peroxidation, have been associated with HF symptoms and disease (71). An excess in reactive oxygen species induces a reduction in cardiac function and myocardial growth, favors adverse remodeling with fibrosis proliferation, and thus participates in HF progression (72). TH is able to affect the antioxidant status, directly (e.g., iodide, I^−^, can act as an electron donor and, as such, be effective as a scavenger of free radicals) or indirectly (e.g., stimulation or inhibition of the activity of antioxidant enzymes and free radical scavengers) (73). The effect of hypothyroidism on oxidative markers is a reduction of antioxidants and an increase in lipid peroxidation (74, 75). Conversely, the cardioprotective effects of TH have been associated with oxidative stress reduction. Specifically, the administration of T3 (2 μg/100 g/day) and T4 (8 μg/100 g/day) by gavage for 26 days in infarcted rats, which showed increased hydrogen peroxide and lipid peroxidation, decreased the reduced glutathione to oxidized glutathione ratio (GSH/GSSG), when compared to controls, reduced reactive oxygen species (ROS) levels, and improved cardiac function (76). Interestingly, in a recent AMI experimental rat model by Ortiz VD et al., the co-administration of carvedilol and TH reduced OxS (with an increase in GSH/GSSG ratio and an attenuation of the increase in NADPH oxidase activity and sulfhydryl group oxidation), and this effect was associated with the improvement of cardiac function. This study has a relevant clinical perspective, considering the potential additive effect of TH treatment over beta-blocker therapy, which is the state-of-the-art treatment of AMI patients (77).

### TH and the Neuroendocrine System

It is well-known that neurohormonal activation represents a central determinant in the genesis and progression of HF. In this context, TH deficiency is frequently reported to reduce sensitivity and responsiveness to catecholamines due to decreases in β1 and β2 adrenoceptor density within the heart (78). In the study of Shao et al., rats with hypothyroidism induced by methimazole had a blunted response to isoproterenol, and this was associated with decreased β1 adrenoceptor (79). However, in the study of Brown et al., although the densities of β1 and β2 adrenoceptors increased in the hypothyroid state, there was an attenuated positive inotropic response to noradrenaline (80). In addition, in the cardiac relationship between TH and the sympathetic system, a potential role is also performed by β3 adrenoceptors, which, differently from β1 and β2, exert a negative inotropic effect (81). The study of Arioglu et al. showed that hypothyroidism induced by methimazole in rats was associated with an increase in the mRNA expression of β3 adrenoceptors and, contextually, the signaling pathway component of these receptors increased (82).

With regard to BNP, there is evidence that TH can modulate BNP release from both atrial and ventricular myocytes and that the plasma level of BNP decreases in hypothyroid rats (83). Moreover, in the study of Liang et al., after T3 treatment, BNP secretion increased 6-fold, BNP mRNA levels 3-fold, and BNP promoter activity 3–5-fold, leading to myocardial hypertrophy in neonatal rat ventricular myocytes (84). Furthermore, the study of Hajje et al. showed that hypothyroidism induced by propylthiouracil induced the expression of the fetal genes for atrial natriuretic peptide (ANP; hormone secreted in response to myocyte stretch in the cardiac atria, similar to BNP in its hemodynamic effects, with vasodilating properties) and BNP; this response was abolished by LT4 treatment (66). The potential effect of the reciprocal regulation of TH and the neuroendocrine system in HF is uncertain. As discussed below, continuous T3 infusion in HF patients induced deactivation of the neuroendocrine system, characterized by a reduction in plasma levels of noradrenaline, NTproBNP, and aldosterone. This neuroendocrine rearrangement may be mediated by a direct effect of TH on these endocrine systems and by an indirect effect due to the improvement in cardiac function (29).

A pathophysiological mechanism that could associate TH and neuroendocrine alterations in HF is that the continuous alterations of these systems may shift the action from adaptive to maladaptive responses. In this context, LT3S may initially be an adaptive process to minimize energy expenditure, but in its persistence can drive a maladaptive mechanism, becoming a factor in HF progression, in line with the negative clinical and prognostic impact in the clinical setting and with the negative structural, histological, cellular, and functional effects experimentally documented in hypothyroid animal models.

### TH and the Epigenetic Way: Chromatin Modifications

THs have a fundamental role in cardiovascular homeostasis, and alterations in TH signaling are associated with cardiac pathophysiology. More recently, it has become more and more evident that the regulatory effects of TH have to be investigated either at the genetic or epigenetic levels in order to individuate new therapeutic tools for the prevention and treatment of TH-dependent cardiac disorders.

Gene regulation occurs under the combined effects of transcription factors and cofactors and their interactions with promoter regulatory regions, and chromatin organization regulates the accessibility of these elements to the DNA.

Furthermore, besides genetic factors, cardiac gene expression is under the control of epigenetic modifications, which do not alter the underlying DNA sequence, either in normal or pathological conditions, being for this reason, reversible. Thus, epigenetic variations can give information beyond genotype, and because of their plastic patterns, they may be very suitable as key factors in the individuation of personalized therapies.

Previous studies have demonstrated the important influence of TH on the pathologic heart, especially on the cardiac MHC phenotype. The effects of TH on gene expression are complex and involve the interaction of several processes, including epigenetic events such as histone modifications and chromatin remodeling (85). Interestingly, transitions between different chromatin settings are the resultant of the balancing between factors that induce and maintain a silent state (corepressors) and elements favoring an active transcriptionally condition (coactivators). Alteration of this equilibrium results in a modification of the transcriptional state (86).

**Figure 3:**
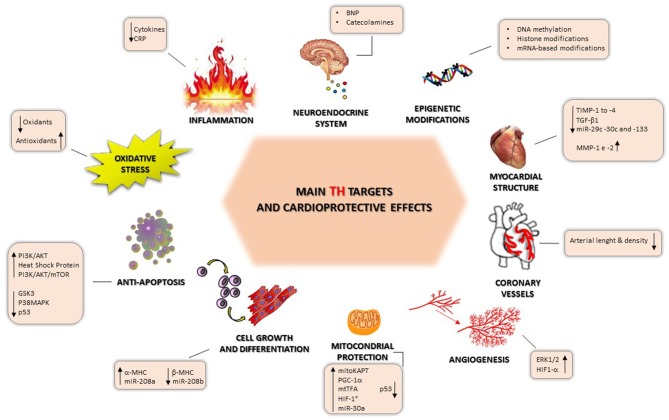
Main thyroid hormone targets and cardioprotection-mediated effects.

In particular, some studies have shown that the antithetical regulation of α and β-MHC genes by TH is under both genetic and epigenetic regulation (87). Both positive and negative regulation at the transcription level are driven by two opposite activities: histone acetylation and deacetylation, mediated, respectively, by histone acetyltransferase (HAT) and histone deacetylase (HDAC) (Figure 3). Furthermore, THs also influence histone methylation in chromatin; however, whereas histone acetylation always has an activating role on chromatin, histone methylation can have either a positive or a negative effect on transcription depending on the methylation site (88).

T3 exerts its effects through nuclear TRs, which bind specific TRE sequences in the promoters of target genes, mediating positive or negative transcription states depending on the TREs involved.

The α-MHC gene is positively regulated by T3 whereas β-MHC is negatively regulated.

The molecular mechanism of T3 action has seen much study: in the absence of T3, TR is associated with nuclear corepressors (i.e., the NCoR/SMRT complex) (89), and, after deacetylation of lysine residues of histones H3 and H4 at TREs, chromatin undergoes structural alterations determining the repression of transcription. After T3 administration, corepressors detach from TRs and are replaced by a set of coactivators promoting histone acetylation (p160/steroid receptor and p300, TRAP/SMCC/Mediator complex) (90, 93). Furthermore, the TR-mediated activation is also associated with the tri-methylation of lysine 4 of histone H3 (H3K4me3) (87), which is considered, together with acetylation, the most conserved marker of gene activation (Figure 3). Interestingly, whereas histone methylation can be associated either with active or repressed genes, try-methylation of lysine 4 of histone H3 is only associated with active genes (86). Therefore, both histone H3 acetylation and H3K4me3 are post-translational indicators of gene expression. In particular, in T3-associated MHC modifications, H3K4me3 can be referred to α-MHC expression, whereas histone H3 acetylation to β-MHC expression.

### TH and miRNA-Based Post-transcriptional Regulation in Cardioprotection

MicroRNAs (miRNAs) are small (20–24 nucleotides), single-stranded, non-coding RNAs that are involved in post-transcriptional modulation of gene expression (Figure 3). miRNAs belong to the non-coding RNA (nc-RNA) family, whose members have a functional role at transcriptional and posttranscriptional levels but are not translated into proteins. Several miRNAs have been individuated as crucial biomarkers of cardiovascular damage, mainly regulating gene expression throughout messenger RNA destabilization (54, 55). Even though the regulatory role of miRNAs in cardiac gene expression is well-documented, the association of miRNAs with regulatory mechanisms involving TH metabolism components in heart disease still has to be clarified (34). For example, it has been observed that, after myocardial infarction, type 3 iodothyronine deiodinase (D3, the main enzyme responsible for cardiac tissue hypothyroidism) and miR-214 are co-expressed in the heart. Since miR-214 is known to be activated in hypothyroidism, it has been hypothesized that it might play an important role in limiting the expression of D3 (35). Furthermore, D3 is considered to be one of the developmentally important genes activated in heart remodeling, associated with T3 reduction in myocardial infarction. Moreover, in pathological hypertrophy, TH directly modulates specific miRNAs, such as miR-208a, as activators, and such as miR-208b, as inhibitors (59). Isoforms of miR-208 are crucially involved in heart contraction and conduction, but pathogenic miR-208 overactivity possesses pro-hypertrophic, pro-fibrotic, and arrhythmogenic properties (60). The administration of specific single-stranded oligonucleotides, termed antagomir, can inactivate pathological miRNAs (61). In particular, targeted silencing of miR-208a, directly involved in the regulation of cardiac stress response, reduces cardiac remodeling and improves cardiac function during heart failure (59, 62).

Recently, the role of miR-27a as a modulator of thyroid hormone signaling has been demonstrated in the β-MHC gene, via TRβ1, in both *in vivo* and *in vitro* studies. In mouse hearts treated with transverse aortic constriction, which results in after-load cardiac hypertrophy, β-MHC gene expression increased whereas TRβ1 was downregulated. Contextually, miR-27a was upregulated, suggesting its association with the developing cardiac hypertrophy in terms of β-MHC regulation via TRβ1 (91).

## Conclusion

The TH system plays a multilevel role in cardioprotection after HF due to the effects on molecular pathways and cardiac function and structure, as confirmed by the large amount of available experimental results. In the clinical setting, the majority of data show altered TH metabolism to have a negative prognostic effect. In view of these findings, the question of whether it is beneficial to treat patients with HF and abnormal TH patterns is not yet completely clear, although very preliminary clinical evidence showed potential positive effects. In this respect, it should be said that the recently updated guidelines of the American Association of Clinical Endocrinologists, in conjunction with the American Thyroid Association, introduced advice for which TH treatment could be considered in patients with HF and TSH levels exceeding the high normality reference limit up to 10 μIU/mL (92). The advice concerns the restoration and maintenance of a normal TH status in HF patients, avoiding over-treatment, which implies the dangerous consequences of pharmacological-induced hyperthyroidism. Large multicenter trials documenting the efficacy and safety of TH treatment in HF are still lacking. Future clinical studies are expected to evaluate which type of hormone (T3 or T4) is more indicated to be administered and by which route, at which dose, and with what timing in order to offer the safest and most effective TH treatment in HF patients. Other possibilities, such as those explored in the inflammatory or oxidative stress scenario (e.g., downregulation of GRK2 expression, use of antioxidants such as N-acetylcysteine, selenium, or vitamin D), may represent alternative/additive therapeutic tools to TH administration, although this research area is still in its infancy. In particular, whether biomarkers belonging to other pathways involved in the thyroid-HF axis (e.g., epigenetic modification, cell growth, and differentiation, myocardial hypertrophy, apoptosis, mitochondrial functioning, neoangiogenesis, and fibrosis) may serve as targets of a replacing or supplementary strategy to TH treatment remains an interesting field for future research.

## Author Contributions

AP: concept definition and writing and final review. FM and CV: writing and review. LS: writing, review, and figures.

### Conflict of Interest

The authors declare that the research was conducted in the absence of any commercial or financial relationships that could be construed as a potential conflict of interest.
